# Effect of Audience Response System on Morbidity and Mortality Conference Engagement

**DOI:** 10.15694/mep.2019.000217.1

**Published:** 2019-11-29

**Authors:** Matthew Zuckerman, Bonnie Kaplan

**Affiliations:** 1 University of Colorado School of Medicine

**Keywords:** Audience Response, Morbidity and Mortality Conference, Computer-Assisted Instruction

## Abstract

This article was migrated. The article was marked as recommended.

**Objectives**: Morbidity and mortality (M & M) conference is a central part of emergency medicine residency training. While audience response systems have become popular in traditional didactic teaching, little research has looked at effects in the unique M & M conference setting. We aimed to evaluate the effects of an audience response system on engagement in emergency medicine morbidity and mortality conference.

**Methods**: An SMS text and internet-based audience response system was integrated into the M & M conference at one site in our emergency medicine residency. Anonymous, quantitative data about respondents use of the system was collected. Conference attendees were also surveyed to assess their evaluation of the ease of engagement in conference, effects of audience response system on engagement in conference and on perceived audience distractions.

**Results**: The number of participants varied by conference and ranged from 37 to 63 respondents who responded from 1 to 21 times per conference (median 2 responses per respondent per conference). Subjects who used the audience response system were significantly more likely to report improved engagement in conference (p = .002). Subjects with more seniority (Assistant, Associate and Full Professors) reported easier engagement with M&M conference in general (p = .003). The audience response system did not result in significant reduction in audience distractions. Unexpected benefits of the audience response system included increased opportunity for engagement as well as quality of feedback for speakers.

**Conclusions**: The integration of an audience response system into our M&M conference resulted in increased engagement and improved quality of speaker feedback. Further research is needed to evaluate effects on learning retention and clarify effects on audience distractions and behavior.

## Introduction

Morbidity and Mortality (M&M) case conference is a unique educational format, combining didactic lecture with an engaged audience discussion by faculty, residents, and students ( Kravet, Howell and Wright, 2006; Seigel *et al.*, 2010). It’s not uncommon that experienced faculty dominate the discussion while junior audience members disengage and become distracted on phones and laptops. We hypothesized that an audience response system would increase audience engagement and address the cause of disengagement.

Audience engagement is dynamic over time, and we hoped that polling the entire audience at certain decision points would provide opportunities for learners to re-engage. Poll Everywhere integrates with PowerPoint to graphically show audience responses in real time. In addition, it allowed faculty facilitators to review comments before addressing them to the speaker. We hoped this would encourage junior learners to feel comfortable asking questions without concern or hesitation. Additionally, the use of faculty facilitators unburdened the presenter from becoming distracted by managing the ongoing audience response system.

Previous audience response systems relied on learners picking up portable keypads which transmitted button presses via radio ( Kay and LeSage, 2009). Newer software and SMS based systems are less expensive than hardware-based systems and avoid issues of hardware failure and loss through theft. Software and SMS based systems allow users to send full text messages and even select parts of an image. 86% of educated cell phone owners use text messaging and about a third of them prefer to interact via text rather than phone call, highlighting the preference many have for text based communication ( Twilio, 2016). For this reason, the Poll Everywhere platform which allows for response via SMS text or web portal on cell phone, tablet, or laptop is gaining in popularity ( Brazil, 2016). While educational innovations often focus on implementation with tech savvy residents and med students, M&M conference attendees vary from digital natives in generation Y to older faculty who may have a harder time installing new apps but have almost all written SMS texts ( Eckleberry-Hunt and Tucciarone, 2011). This study evaluated the use of technology, such as Poll Everywhere, to engage participants in M&M conference. We hypothesized that by using Poll Everywhere we would increase participants feeling of engagement in conference. In addition, we tested our belief that junior learners were less comfortable than senior faculty in engaging in M&M conference considering medical education hierarchy. We also looked at whether those that used the technology were more distracted. Finally, we evaluated whether participants felt that this technology made audience response easier.

## Development Process

We had informal discussions with a variety of stakeholders (senior and junior faculty, residents, residency leadership) about our concern that this portion of conference was frequently dominated by a subset of senior faculty. It appeared that more junior members of the department may be reluctant to participate out of intimidation or a perceived difficulty. Additionally, there was concern that the above factors created an incentive for audience members to disengage and work on other tasks, missing out on the educational objective of M&M conference. Some stakeholders suggested banning distracting mobile devices and laptops from conference, but we felt that this didn’t address the critical issues that lead to audience disengagement. Adult learning theory says that knowledge is constructed rather than transmitted, and prompting learners to formulate the answer to an audience survey outperforms merely telling them the answer ( Gousseau, Sommerfeld and Gooi, 2016). Additionally, M&M is a key opportunity for reflection and experiential learning; even audience members not involved in the case can reflect “Could this have been me” and learn from others decisions ( Gregor and Taylor, 2016).

We ultimately selected the Poll Everywhere audience response platform.

## Methods

### Participants

Participants were 35 academic Emergency Medicine (EM) physicians who responded to an optional survey. The participants different “roles” in the department included 19 residents, 1 instructor, 7 assistant professors, 6 associate professors, and 1 professor and 1 who didn’t answer this question. The participants are all part of the academic Department of Emergency Medicine attending M&M conference at the University of Colorado School of Medicine. Additional participants attended conference and used the Poll Everywhere system but chose not to respond to the follow up survey.

### Study setting

We implemented this study during the weekly M&M conference at a large academic emergency medicine residency program. Participants included attendees of the conference which include faculty, staff, house officers, medical students, undergraduate students, nurses, advance practice providers, and guests. Conference alternates weekly between two campuses and the study was only implemented at one of them. This provided a control of conferences where the audience response system was not available.

### Study protocol

We integrated an anonymous audience response system (Poll Everywhere, Chicago, IL) into our M&M conferences at one site. The software allows respondents to join via web portal or SMS text message. Audience members could interact via one of two ways. They could answer multiple choice surveys in real time. Cumulative totals of survey responses are shown on the screen in real time. Additionally, between audience surveys, audience members may send in text comments. To avoid distracting the speaker, these comments were reviewed by a faculty moderator and addressed to the speaker. Comments that were already addressed by the speaker or were duplicative or not relevant were not addressed to the speaker. During one session, we did not use audience surveys and only included text comments. This allows for comparison between audience response design.

The department received no material support from Poll Everywhere and none of the authors have any conflict of interest to report.

### Measures

To assess audience impression of this system we analyzed responses using a descriptive and quantitative analysis. In addition to raw response data, we also conducted a survey of attendees of our residency weekly M&M conference. We designed an 11-item survey instrument to assess their impression of M&M, specifically addressing engagement and freedom to participate. The survey was administered using a web-based survey tool (Google Forms, Mountain View, CA) and is attached (Supplementary File 1). If a respondent did not answer all questions, their complete answers were included. The Colorado Multiple Institutional Review Board approved this study as exempt and not human subjects research as it seeks to assess a pre-existing program (Application 17-1662).

### Data analysis

All the results were imported from google forms into an excel spreadsheet. The data was then coded and uploaded into SPSS for analysis. Analysis was done to test the hypotheses as previously stated.

## Results

Number of participants varied by conference and ranged from 37 to 63 respondents, excluding one outlier with only 5 respondents. This outlier represents a conference where we did not include multiple choice surveys and only allowed for text questions/feedback. Respondents interacted with the system from 1 to 21 times during each conference, with a median response of 2 times.

To answer the research question “Is there a difference in participant engagement between those who used Poll Everywhere and those who didn’t,” a nonparametric test, the Mann Whitney U test, was conducted. The participants who used Poll Everywhere were found to have significantly more engagement than those who didn’t use Poll Everywhere ( *U* =19, *p* =0.002).

To answer the research question “Is there a difference between participants’ role in the department and their comfort level with engaging in M&M,” a nonparametric test, Kruskal Wallis H test was conducted. Participants were asked to pick their “role” in the department. In analysis we combined residents with instructors, considering they are recent graduates of residency programs and the assistant, associate and professor roles. They then had to answer from 1 to 4 on a Likert scale on comfort in participation in conference (1 not comfortable, 4 very comfortable). Subjects with more seniority reported easier engagement with M&M conference in general (p = .003).

To answer the research question “Is there a difference in participant distraction if they use Poll Everywhere,” a nonparametric test, the Mann Whitney U test, was conducted. Participants were divided into use versus no use. The dependent variable of distraction was divided into a 3-point Likert scale with rarely (1), sometimes (2), or frequently (3). There was no significant effect found on use of Poll Everywhere and self-reported distraction during conference.

Finally, a nonparametric test, the Mann Whitney U test, was conducted to see if those that used Poll Everywhere felt participating in conference was significantly easier. A statistically significant relationship was found between Poll Everywhere use and participants thoughts that participating in conference was easier, ( *U =*22, *p* =0.02). Thus, those that used Poll Everywhere felt that it was easier to engage in conferences.

## Discussion

Utilizing audience feedback to allow real time engagement with learners and faculty is feasible and effective in an M&M environment. During our study, learners at a variety of levels were able to increase their engagement in the conference using this technology.

The audience response system provides a comfortable and easy way to make sure all those in the educational hierarchy feel they can participate in conference. Poll Everywhere allowed for an anonymous channel for commentary and engagement. This may explain our finding that the system made participation in conference easier and increased users perception of audience engagement.

In addition to the measured outcomes, there were several secondary benefits. We found that audience members used the technology to give real time feedback to the speaker about their presentation style. Speakers at our conference usually receive feedback via mandatory audience surveys completed minutes to weeks after conference. These surveys often do not contain useful and actionable feedback for the speaker. The spontaneous real time feedback provided via the audience response system was specific, concrete, and audience initiated rather than required. We compiled this feedback and gave it to speakers to improve their speaking skills.

We reasoned that increasing engagement would keep audience members from becoming distracted from the talk. We did not find a reduction in reported distractions, however further study with larger sample sizes may clarify this relationship. Anecdotally, we found that the survey questions we administered for each case provided an opportunity for re-engagement of distracted audience members. We saw people close laptops and stop peripheral conversations to respond to the multiple-choice surveys posed to the audience (e.g. “What would you do next: A. CT scan B. X-ray C. Consult surgery”). As audience members pulled out their phones to respond to these surveys, the distracted audience members next to them put away their distractions and re-engaged. Thus, the technology may actually help maintain overall engagement by allowing re-engagement.

Senior faculty often perceive no barrier to raising their hand and offering opinions and scholarly resources on the case at hand. However, junior learners sometimes feel less secure. The audience response system increased participation from medical students and residents by allowing anonymous contributions that were moderated. Learners were able to submit comments immediately without having to keep their hand up.

During a conference length of 90 minutes we typically have time to discuss 3 cases with about 4-5 comments or questions per case. That allows for a maximum of 15 participants out of an average attendance of 70. Using the audience response system, we found that 37-63 audience members participated from 1 to 21 times (median of 2 responses). The variability suggests heterogeneity in audience utilization. We did not find that the option of anonymous text feedback decreased traditional engagement from the audience members who continued to raise their hand to engage. The additive effect meant that more people were engaging with conference more times than ever before. Conferences with multiple choice survey questions had more respondents overall and more free text comments than conferences with only free text conferences.

Finally, audience members commented that they appreciated the way survey questions forced responses before a case was fully completed. Many felt that M&M conference sometimes encourages a retrospective bias where each audience member believes they would have predicted the unexpected result or outcome. By forcing audience members to predict the case course early on, we reflected the reality of emergency medicine which often requires making a decision before all the information is known.

## Limitations

We acknowledge that this study has limitations. The study occurred at one of two conference sites with learners from one residency program and medical school. The study was underpowered to detect subtle changes and to allow for subgroup analysis (e.g. medical students vs faculty). This study was not blinded. This study did not use a control group, although we did attempt to compare attendees who had been exposed to the intervention with those that had not.

## Conclusions

In conclusion, we view the implementation of an audience response system during morbidity and mortality conference to be a success. Audience members were more engaged and given significantly more opportunity for participation. Anonymous real time responses allowed participation by learners and faculty who might otherwise not participate. An unexpected benefit was the real time feedback given to presenters. We received positive feedback from audience members and speakers about the benefits of the audience feedback system. This is possibly because an engaged audience is generally more satisfying to be in and to speak to. In the future we plan to evaluate whether this intervention improved knowledge retention or changed behaviors.

## Take Home Messages

• Implementing an audience response system during morbidity and mortality conference is feasible.
• More senior participants find participation in conference to be easier; however, an audience response system can make participation easier and increase engagement overall.
• An audience response system can provide opportunities for real time feedback on the speaker.

## Notes On Contributors

Matthew Zuckerman is an Assistant Professor at the University of Colorado School of Medicine. He is a medical toxicologist and emergency medicine physician with a special interest in using technology in education. ORCID: https://orcid.org/0000-0001-9576-4203

Bonnie Kaplan is Program Director of the Denver Health Residency in Emergency Medicine.
