# Supplementary material for: Effect of Audience Response System on Morbidity and Mortality Conference Engagement
Source: MedEdPublish (2016). 2019 Nov 29;8:217. [Version 1] doi: 10.15694/mep.2019.000217.1 (PMC10712458; doi:10.15694/mep.2019.000217.1)
Supplement: Peer review history

# Reviewer report: Reviewer response for version 1

**DOI:** 10.21956/mep.19927.r30222

**Published:** 2020-01-27

**Authors:** Julie Hunt

**Affiliations:** 1 Lincoln Memorial University

This review has been migrated. The reviewer awarded 4 stars out of 5 Audience response devices have proven helpful in increasing engagement and recall of facts in numerous fields, so it's not surprising to hear of their success at M&M conference as well. Although their results are not surprising given the existing body of work on the topic, the authors do a good job of discussing their particular findings. Educators not currently using audience response systems except for didactic classroom training should read this article--and other articles about the myriad uses for these systems--to better understand their diverse applications.

Reviewer Expertise:

NA

No decision status is available

# Reviewer report: Reviewer response for version 1

**DOI:** 10.21956/mep.19927.r30221

**Published:** 2019-11-29

**Authors:** P Ravi Shankar

**Affiliations:** 1 American International Medical University

This review has been migrated. The reviewer awarded 4 stars out of 5 I enjoyed reading this article about the implementation of an audience response system (ARS) at morbidity and mortality conferences in an academic emergency medicine department. ARS is now being more widely implemented in medical and health education and modern software offer the capability of offering the same through cell phones or tablets. I agree with the authors that offering an anonymous ‘voice’ will encourage participation of junior attendees. I have a few questions for the authors? Why did the authors select the poll everywhere platform? There are similar systems with similar capabilities. Were there any specific reasons for choosing Poll Everywhere? How many questions were administered per conference? How did the audience submit questions and comments for the speaker? I would be interested in knowing who all can attend the conference? The authors do mention guests and advance practice providers among others. The article will be of interest to all educators.

Reviewer Expertise:

NA

No decision status is available
